# Sensitivity Analysis of Left Ventricle with Dilated Cardiomyopathy in Fluid Structure Simulation

**DOI:** 10.1371/journal.pone.0067097

**Published:** 2013-06-25

**Authors:** Bee Ting Chan, Noor Azuan Abu Osman, Einly Lim, Kok Han Chee, Yang Faridah Abdul Aziz, Amr Al Abed, Nigel H. Lovell, Socrates Dokos

**Affiliations:** 1 Department of Biomedical Engineering, Faculty of Engineering, University of Malaya, Kuala Lumpur, Malaysia; 2 Department of Medicine, Faculty of Medicine Building, University of Malaya, Kuala Lumpur, Malaysia; 3 Department of Biomedical Imaging, University Malaya Research Imaging Centre, Faculty of Medicine, University of Malaya, Kuala Lumpur, Malaysia; 4 Graduate School of Biomedical Engineering, University of New South Wales, Sydney, New South Wales, Austrailia; University Hospital of Würzburg, Germany

## Abstract

Dilated cardiomyopathy (DCM) is the most common myocardial disease. It not only leads to systolic dysfunction but also diastolic deficiency. We sought to investigate the effect of idiopathic and ischemic DCM on the intraventricular fluid dynamics and myocardial wall mechanics using a 2D axisymmetrical fluid structure interaction model. In addition, we also studied the individual effect of parameters related to DCM, i.e. peak E-wave velocity, end systolic volume, wall compliance and sphericity index on several important fluid dynamics and myocardial wall mechanics variables during ventricular filling. Intraventricular fluid dynamics and myocardial wall deformation are significantly impaired under DCM conditions, being demonstrated by low vortex intensity, low flow propagation velocity, low intraventricular pressure difference (IVPD) and strain rates, and high-end diastolic pressure and wall stress. Our sensitivity analysis results showed that flow propagation velocity substantially decreases with an increase in wall stiffness, and is relatively independent of preload at low-peak E-wave velocity. Early IVPD is mainly affected by the rate of change of the early filling velocity and end systolic volume which changes the ventriculo:annular ratio. Regional strain rate, on the other hand, is significantly correlated with regional stiffness, and therefore forms a useful indicator for myocardial regional ischemia. The sensitivity analysis results enhance our understanding of the mechanisms leading to clinically observable changes in patients with DCM.

## Introduction

DCM is associated with complex remodeling of one or both ventricles, resulting in a change of the ventricle shape and the architecture of the myocardium fibers. The ventricular shape changes from an elliptical to a more spherical form [1], [2]. In addition, patients with DCM may have a stiffer myocardial wall, caused by increased myocardial mass and alteration in the extracellular collagen network [3]. DCM secondary to ischemic heart disease, however, may have different areas of viable myocardium and non-viable myocardium. The viable myocardium would be contracting normally while the non-viable myocardium contracts weakly. Indeed, Parodi et al. [4] observed a more heterogeneous transmural blood flow distribution in patients with ischemic DCM and a higher percentage of fibrosis in these patients in their clinical studies despite having a similar mechanical compensation and global hemodynamics,.

The structural ventricular remodeling processes associated with DCM, including chamber enlargement, alterations in shape and decreased wall compliance significantly affect the intraventricular filling dynamics. Experimental study [5] has reported that the transmitral filling pattern revealed a progression of the heart disease due to a gradual decrease in wall compliance and increase in LV pressure. The impaired relaxation filling pattern indicates an early stage of diastolic dysfunction (Grade I), followed by a more severe pseudonormal filling pattern (Grade II). The restrictive filling pattern (Grade III-IV) implies the most advanced disease stage [6]. Extensive clinical studies have investigated the difference between normal subjects and DCM patients with regards to the intraventricular fluid dynamics (e.g. flow propagation velocity [7], intraventricular pressure difference (IVPD) [8], [9], vortex area [10], [11], vortex intensity [11]) as well as myocardial wall mechanics (e.g. principal strain [12] and strain rate [13], [14]) using different imaging modalities, including phase contrast magnetic resonance imaging (MRI) [15], [16], color M mode echocardiography [7], contrast echocardiography [10] and tissue Doppler imaging (TDI) [14], [17].

One limitation of these studies is that controversial findings are often reported, as there is a high variability among patient characteristics, and it is difficult to ascertain the effect of individual physiological variables on the parameters of interest [7]. For example, Stoylen et al. [14] found an increase in flow propagation velocity in patients with moderate hypertrophy, as opposed to other studies which demonstrated reduced flow propagation velocity in patients with reduced diastolic function [18]. Furthermore, limitations in the measurement methods also contribute to inconsistent findings. For instance, strain measurements using Doppler-derived techniques face the problem of angle dependency [19] while IVPD measurements using color Doppler M-mode images may yield high error when applied to severely dilated ventricles [8].

To overcome this problem, computational fluid dynamics (CFD) and computational structural mechanics (CSM), which quantify the spatial and temporal distributions of the velocity, pressure, stress and strain in the heart, have emerged as reliable tools in enhancing our understanding of the pathophysiology and progression of structural heart disease. Although numerous models of ventricular biomechanics for the ventricles have been published [20], they have not been widely used in the study of diseased states. One of the few studies is the geometry-prescribed model by Baccani et al. [21], who analyzed the vortex dynamics in an axisymmetrical left ventricle (LV) with DCM during filling. It was observed that geometrical alteration of the LV in DCM decreases flow propagation velocity which may lead to the formation of apical thrombus. In another study, Loerakker et al. [11] developed an axisymmetrical fluid dynamics model of the LV, coupled to a lumped parameter model of the complete circulation, to investigate the influence of a ventricular assist device on the vortex dynamics in a ventricle with dilated cardiomyopathy. On the other hand, Choi et al. [22] found that an increase in wall thickness decreases strain in the mid-wall region of the ventricle, while leaving the subendocardial strain values relatively unchanged. They suggested that this result may explain the experimental observations by Zabalgoitia et al. [23], who observed normal endocardial shortening in patients with concentric hypertrophy despite a decrease in mid-wall systolic shortening. The major limitation of the previous works is that interaction between blood flow and myocardial wall deformation, which significantly affects the various parameters of interest, has not been taken into account.

The aim of the present study is twofold: (i) to investigate the effect of idiopathic and ischemic DCM on intraventricular fluid dynamics (i.e. vortex area, vortex intensity, average vorticity, flow propagation velocity, early peak IVPD, late peak IVPD) and myocardial wall mechanics (average wall stress, longitudinal, circumferential and radial strain rates) using a 2D axisymmetrical fluid structure interaction (FSI) model; and (ii) to investigate the individual effect of parameters related to DCM, i.e. peak E-wave velocity, end systolic volume (ESV), wall stiffness and sphericity index (SI) on intraventricular fluid dynamics and myocardial wall mechanics during LV filling. In this study, the idiopathic and ischemic DCM ventricles are simulated by increasing ESV (to simulate chamber enlargement), wall stiffness (globally for the idiopathic DCM case and at the apical region for the ischemic DCM case) and SI of the ventricle under baseline normal condition.

## Methods

### I. Model Framework

**Figure 1:**
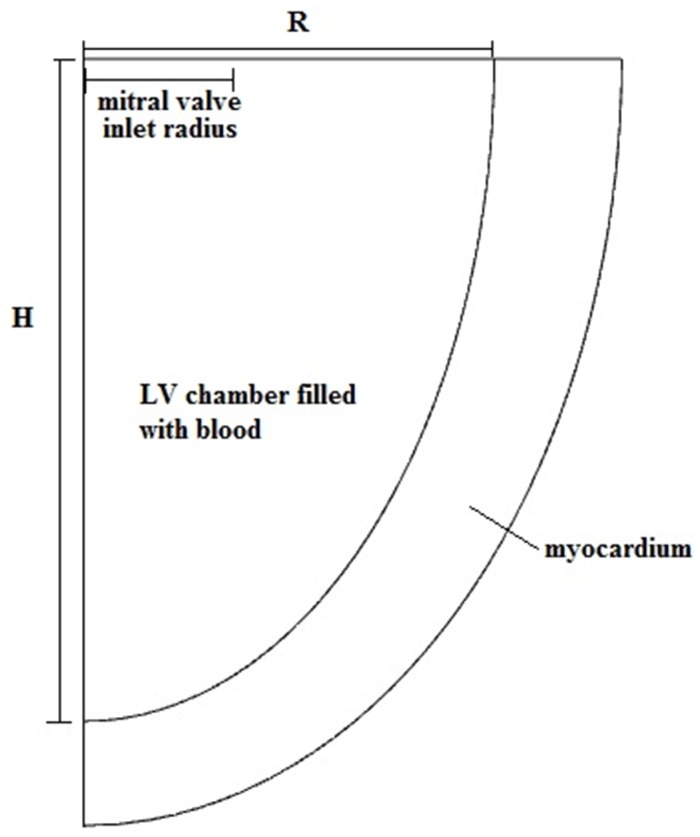
Basic geometry of the LV in the computational model.

The LV is modeled as an axisymmetric half-truncated prolate ellipsoid. The SI of the left ventricle is calculated from the ratio of the ventricular radius (R) at the base equatorial plane to the ventricular height (H) measured from the base to the apex, as shown in Figure 1. At the ventricular base, the mitral valve is modeled as a circular inlet with a fixed ventriculo:annular ratio while the aortic valve is closed throughout the filling phase. Ventriculo:annular ratio refers to the ratio of the LV radius to the radius of the mitral valve (or mitral annular ring).

Blood was assumed to be incompressible and Newtonian, with a viscosity of 0.0035 Pa.s and density of 1050 kg/m^3^. A hyperelastic wall with a density of 1366 kg/m^3^ was used to represent the ventricular muscle in the passive state. A first-order Ogden constitutive relation [24] was used to represent the hyperelastic myocardium:
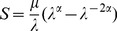
(1)where S (kPa) is the nominal stress, λ represents the principal stretch, whilst µ and α are material parameters.

Ventricular wall thickness decreased from the base to the apex, being 11 mm at the base and 9 mm at the apex. The basal wall was held fixed in all directions, whilst other parts of the wall were allowed to move freely. To simulate transmitral flow, a uniform velocity profile was applied at the inlet boundary representing the mitral valve. The time-varying profile of the filling phase was comprised of the E-wave, diastasis and A-wave components, representing the three main phases of the transmitral inflow. When blood filled the ventricular chamber, the ventricular wall deformed accordingly, depending on the load imposed by the fluid and the wall properties.

### II. Theoretical Framework

The quantitative blood flow dynamics were solved using the continuity (Eq. 2) and incompressible Navier-Stokes equations (Eq. 3):
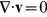
(2)

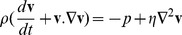
(3)where **v** (m/s) is the fluid velocity vector with respect to the global (fixed) coordinate system, ρ (kg/m^3^) is the fluid density, p (Pa) is the pressure and η (Pa.s) is the fluid viscosity.

The coupling between the fluid and the myocardial wall is taken into account at the fluid-solid interface through the following relationships:
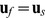
(4)

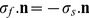
(5)where **u** *_f_* **, u** *_s_* (m) represents the displacements, **σ** *_f_*, **σ** *_s_* (Pa) represents the stress tensors of the blood and myocardial wall respectively at the fluid solid interface, and **n** represents the unit normal to the fluid-solid boundary.

### III. Model Settings

**Figure 2:**
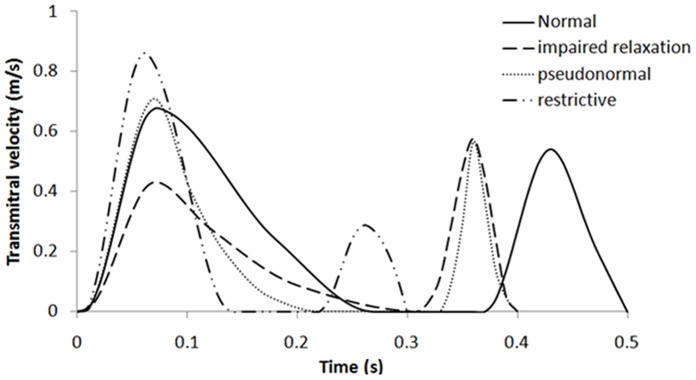
Temporal waveforms of the inlet velocity for normal, impaired relaxation, pseudonormal, and restrictive filling patterns. For each of the filling pattern, E-wave refers to the first wave while A-wave refers to the second wave.

Three cases were considered: normal, idiopathic and ischemic DCM. Three filling patterns (impaired relaxation, pseudonormal and restrictive filling) were simulated for each DCM case. In total seven different conditions are simulated. In order to appropriately simulate the various conditions, ESV, filling velocity (including peak E-wave velocity, E/A ratio and E-wave deceleration time, as shown in Figure 2), valve ratio, SI, and wall stiffness were varied accordingly to closely match the clinical measurements reported in the literature [8], [25], [26] and EAE/ASE recommendations by Nagueh et al [27].

**Figure 3:**
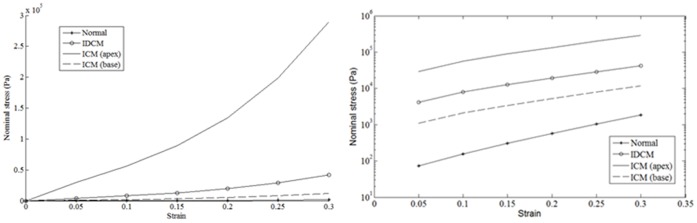
Relationship between nominal stress and strain for the normal, idiopathic DCM and ischemic DCM (basal and apical region) models. Semi-log plot (y-axis) on the right.

We adapted the method proposed by Watanabe et al. [28] in selecting suitable material parameters to fit the Ogden constitutive equation (Eq. 1) for the normal, idiopathic DCM and ischemic DCM conditions (Table 1). Parameter values were determined so that a predetermined end diastolic pressure could be achieved for each condition. The wall stiffness was constant throughout the whole ventricular wall for the normal and idiopathic DCM models. On the contrary, it was much higher in the apical region of the ischemic DCM model (60% of the total wall region) compared to other areas of the LV in order to simulate myocardial infarction. Figure 3 shows the relationship between nominal stress and strain for the normal, idiopathic DCM and ischemic DCM models.

**Table 1:** List of parameter values for normal, idiopathic DCM (IDCM) and ischemic DCM (ICM) conditions.

LV	ESV (mL)	EDV (mL)	IV (mL)	Valve ratio	SI	EDP (mmHg)	E peak (m/s)	A peak (m/s)	DT (ms)	E/A	µ (N/m^2^)	α
Normal	50	120	70	0.6	0.52	9.5	0.7	0.56	200	1.25	39.3	15.7
IDCM (IR)	150	194	44	0.4	0.62	20.3	0.45	0.6	250	0.75	3310	10.7
IDCM (PS)							0.74	0.59	160	1.25		
IDCM (RE)							0.9	0.3	70	3.0		
ICM (IR)	150	194	44	0.4	0.62	20.3	0.45	0.6	250	0.75	23705 (I) 843.2 (NI)	10.5 (I) 11.1 (NI)
ICM (PS)							0.74	0.59	160	1.25		
ICM (RE)							0.9	0.3	70	3.0		

IR = impaired relaxation, PS = pseudonormal, RE = restrictive. ESV = end systolic volume, EDV = end diastole volume, IV = total inflow volume, SI = sphericity index, EDP = end diastolic pressure, E peak = peak E-wave velocity, A peak = peak A-wave velocity, DT = E-wave deceleration time, E/A = ratio of the peak E-wave velocity to the peak A-wave velocity, µ and α = material constants of the Ogden constitutive equation, NI = non-ischemic, I = ischemic.

### IV. Sensitivity Analysis

The effects of a two-fold increase in the baseline normal value of peak E-wave velocity, ESV, wall stiffness and SI on intraventricular fluid dynamics and myocardial wall mechanics were investigated.

Average vorticity, 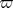
(1/s), was defined as the mean value of the out-of-plane vorticity 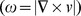 in the LV. On the other hand, quantitative evaluation of the vortex area (Γ_A_) and intensity Γ_I_ are carried out by first identifying the vortical regions in the LV using the λ_2_ criterion proposed by Jeong et al. [29]. The eigenvalues (λ_1_, λ_2_, λ_3_) of the tensor S^2^+ Ω^2^ are computed, where S and Ω are the symmetric and antisymmetric parts of the velocity gradient tensor 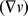 respectively. Assuming that 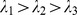, vortical regions are identified as regions with 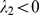. Vortex intensity, Γ_I_ is thus calculated as
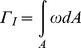
(6)where A (m^2^) represents the area of the vortical regions. The vortex area, Γ_A_, was defined as the total area of all vortical regions. Flow propagation velocity, V_p_ (m/s), was calculated as the average travelling velocity of the peak E-wave mid-way into the LV axis from the inlet.

### V. Effect of Infarct Location

We have extended our investigation to the effect of lateral infarction by modifying the model settings used for the ischemic DCM model. The same percentage of infarction (60% of the total wall region) as well as material properties (i.e. nominal stress-strain relationship) were used for the ischemic- and the non-ischemic region as per the ischemic DCM model. The infarct location was shifted from the LV apex to the middle of the LV to represent lateral infarction.

## Results

### I. Comparison between Normal, Idiopathic DCM and Ischemic DCM Conditions

#### Intraventricular flow distribution

**Figure 4:**
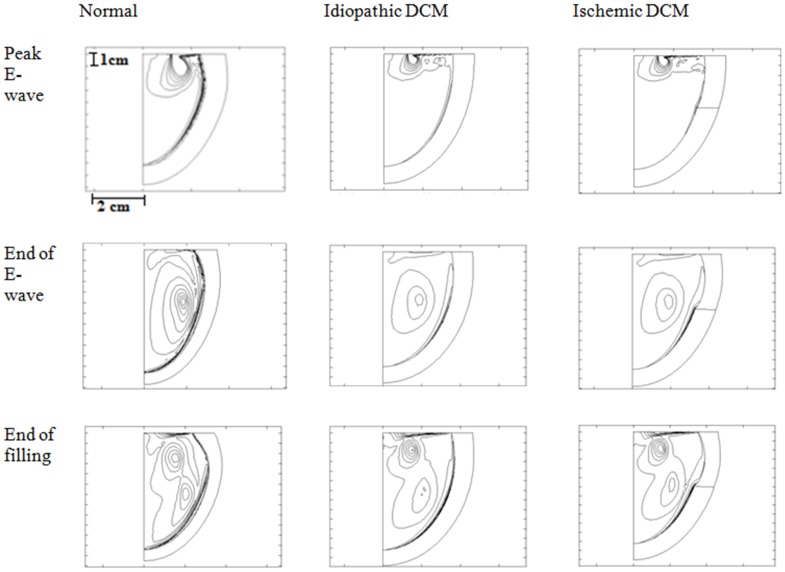
Instantaneous vorticity (contour plot) in normal LV, idiopathic DCM (impaired relaxation) and ischemic DCM (impaired relaxation) during peak E-wave, end of E-wave and end of filling phases. Vorticity levels: from 5 to 100, step 10.

Figure 4 shows the simulated vorticity contour plot for the normal LV, idiopathic DCM (impaired relaxation) and ischemic DCM (impaired relaxation) conditions during the peak E-wave, end of E-wave and end of filling phase. In all cases, as blood flows across the mitral orifice to the larger ventricular chamber, it decelerates and thus pressure increases. The higher pressure downstream opposes the incoming blood flow and redirects it, forming a recirculation structure known as vortex ring. Meanwhile, the vortex induces the formation of boundary layers at the ventricular wall due to the viscous adherence condition at the wall. The vortex creates local velocity gradient along the wall and this perturbation gives rise to a vortex-induced separation of the boundary layer, which rotates in the opposite direction. At the end of the E-wave, the vortex has travelled mid way into the LV axis in all cases. A secondary vortex ring induced by the atrial contraction can be observed in all simulations at the end of filling. Notable differences can be observed between the normal and the DCM cases in terms of the vorticity distribution (Figure 4). However, the vorticity distribution in the idiopathic DCM condition is similar to that of ischemic DCM. It can be seen that the vortices in the DCM cases have a more spherical shape compared to the normal condition, where they are more elongated in shape. Furthermore, the relative area occupied by the vortices compared to the total LV area is much higher in the normal condition. Although the propagation velocity of the vortices is similar among the three cases, the vortex did not reach the apical region during end filling in both DCM cases, in contrast to that of the normal case, due to differences in their LV size. Quantitatively the normal LV has the highest 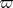, followed by the ischemic and idiopathic DCM cases (Table 2). Among the different filling patterns the restrictive filling pattern yields the highest 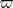, followed by the pseudonormal and impaired relaxation cases. Both DCM conditions exhibit a higher Γ_A_ as compared to the normal LV condition. On the other hand, DCM with a restrictive filling pattern has the highest Γ_I_, followed by normal LV condition, DCM with a pseudonormal filling pattern and lastly DCM with an impaired relaxation pattern. Flow propagation velocity is higher in the normal LV compared to the DCM. Meanwhile, among the various filling patterns, DCM with a restrictive filling pattern exhibited the greatest flow propagation velocity, followed by the pseudonormal and impaired relaxation cases.

**Table 2:** Quantitative comparison of various intraventricular fluid dynamics and myocardial wall mechanics measurements for normal, idiopathic and ischemic DCM with impaired relaxation, pseudonormal and restrictive filling patterns.

Measure-ment	Normal	Idiopathic DCM			Ischemic DCM		
		Impaired	Pseudo	Restrictive	Impaired	Pseudo	Restrictive
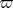 (1/s)	8.90	3.47	5.52	7.29	3.66	5.74	7.49
Γ_A_ (cm^2^)	2.21	2.26	2.72	2.71	2.31	2.64	2.67
Γ_I_ (m^2^/s)	0.011	0.006	0.010	0.013	0.006	0.010	0.013
V_p_ (m/s)	0.445	0.150	0.286	0.357	0.098	0.259	0.357
IVPD_E_ (mmHg)	0.833	0.548	0.828	1.17	0.543	0.841	1.14
IVPD_L_ (mmHg)	0.644	0.908	0.979	0.306	0.788	0.871	0.196
vMS (kPa)	0.844	3.06	3.60	3.682	2.849	3.57	3.667
SR_L_	0.655	0.21	0.208	0.281	0.230	0.231	0.308
SR_C_	0.509	0.154	0.152	0.207	0.0756	0.0754	0.1002
SR_R_	−0.574	−0.246	−0.244	−0.329	−0.165	−0.166	−0.221

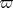
 = average vorticity, ΓA = vortex area, ΓI = vortex intensity, Vp = flow propagation velocity, IVPDE = early peak intraventricular pressure difference (IVPD), IVPDL = late peak IVPD, vMS = average von Mises stress, SRL = average longitudinal strain rate, SRC = average circumferential strain rate, SRR = average radial strain rate.

#### Intraventricular pressure, wall stress and strain distribution

**Figure 5:**
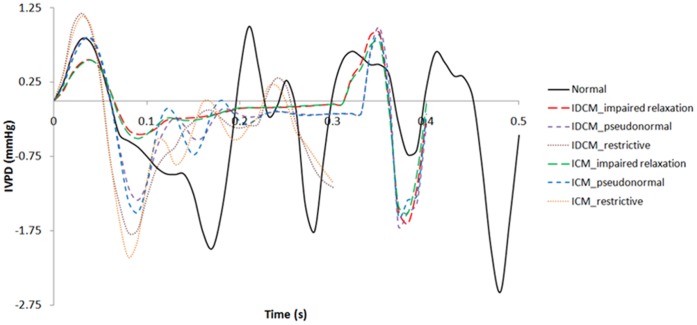
Temporal waveforms of the intraventricular pressure difference (IVPD) for normal, idiopathic and ischemic DCM with impaired relaxation, pseudonormal and restrictive filling patterns.

Figure 5 illustrates the temporal waveforms of the IVPD for normal, idiopathic DCM and ischemic DCM conditions with different filling patterns. Generally, normal LV shows the greatest fluctuations throughout the filling phase. It can be observed that early peak IVPD occurs during the acceleration phase of E-wave (peak acceleration) in all cases. DCM conditions with a restrictive filling pattern have the highest early peak IVPD, followed by DCM with a pseudonormal filling pattern, normal LV, and lastly DCM with impaired relaxation. On the contrary, among the different conditions, DCM with a restrictive filling pattern exhibited the lowest late peak IVPD.

**Figure 6:**
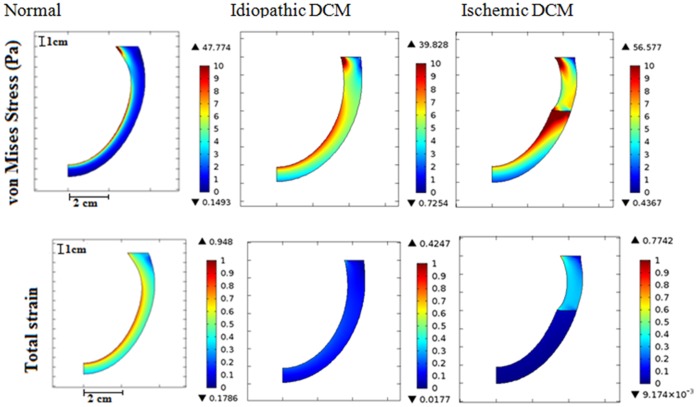
von Mises stress (top) and total strain (bottom) distribution for (a) normal, (b) idiopathic DCM (impaired relaxation) and (c) ischemic DCM (impaired relaxation) during end of the filling phase.

Figure 6 shows the von Mises stress (vMS) and strain distribution in the normal LV, idiopathic DCM (impaired relaxation) and ischemic DCM (impaired relaxation) cases during end of the filling phase. Our findings indicate a decreasing transmural stress distribution from the endocardial to the epicardial wall in all cases during the filling phase. On the other hand, average transmural stress at the mid-height level is higher compared to the apical region. Ischemic DCM demonstrates high wall stress at the border zone between the ischemic and the non-ischemic regions. Compared to the normal LV case, the average wall stress for the DCM conditions is much higher (Table 2). On the contrary, normal LV exhibits the greatest strain and strain rate in all directions, i.e. longitudinal, circumferential, and radial directions (Figure 6 & Table 2). The ischemic region in the ischemic DCM condition has much lower strain level as compared to other regions along the myocardial wall (Figure 6).

#### Pressure volume relationship

**Figure 7:**
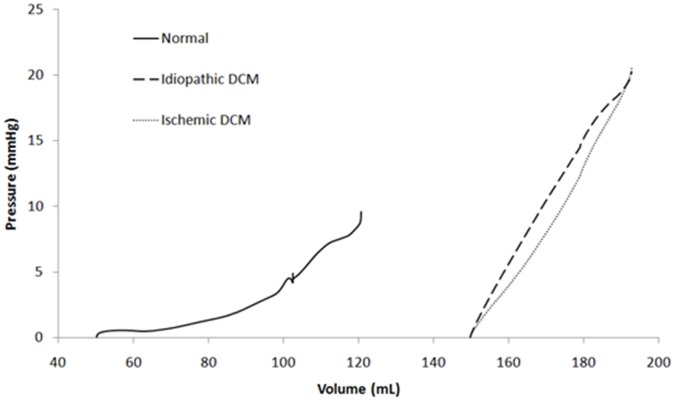
Pressure volume relationship for normal, idiopathic and ischemic DCM cases throughout the filling phase.

Figure 7 shows the LV pressure-volume (PV) curve obtained throughout the filling phase in all cases. It is shown that the PV curve is shifted to the right in the DCM cases, with an increase in slope. Although both DCM cases have the same initial and end PV points, they traverse a slightly different path, with the ischemic DCM appearing to be more compliant within the range used in the present study.

### II. Sensitivity Analysis

**Figure 8:**
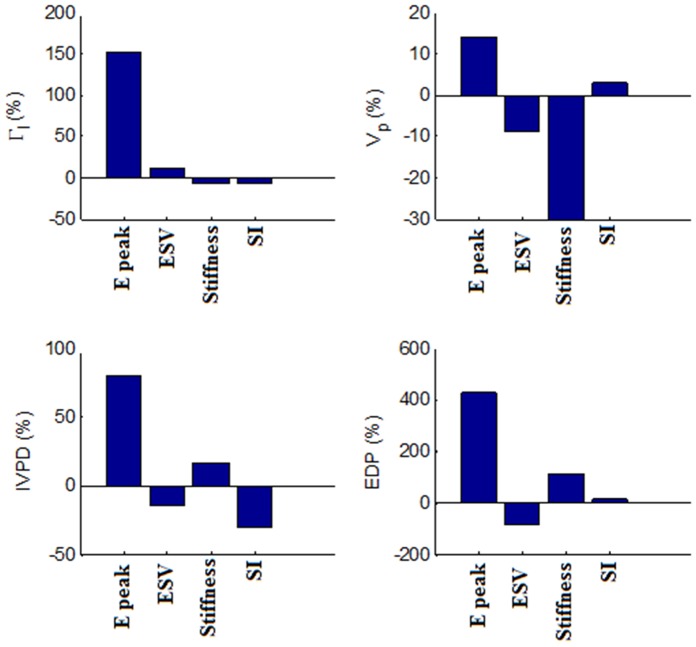
Effects of DCM parameters on fluid dynamic measurements. The percentage change in the vortex intensity (Γ_I_), flow propagation velocity (V_p_), early peak intraventricular pressure difference (IVPD) and end diastolic pressure (EDP) with a 2X increase in the peak E-wave velocity (E peak, baseline value = 0.7 m/s), end systolic volume (ESV, baseline value = 50 mL), wall stiffness (Stiffness, baseline value obtained from the normal condition) and sphericity index (SI, baseline value = 0.52).

**Figure 9:**
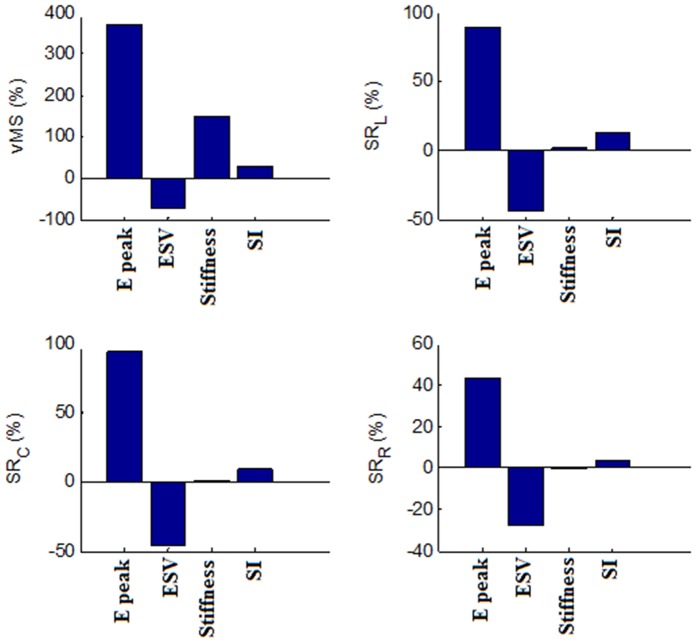
Effect of DCM parameters on wall mechanics measurement. The percentage change in the average von Mises stress (vMS), longitudinal strain rate (SR_L_), circumferential strain rate (SR_C_) and radial strain rate (SR_R_) with a two-fold increase in the peak E-wave velocity (E peak, baseline value = 0.7 m/s), end systolic volume (ESV, baseline value = 50 mL), wall stiffness (Stiffness, baseline value obtained from the normal condition) and sphericity index (SI, baseline value = 0.52).

Figures 8 and 9 illustrate the percentage change in Γ_I_, V_p_, early peak IVPD, EDP, as well as vMS, SR_L_, SR_C_, and SR_R_ of the myocardial wall with a two-fold increase in the peak E-wave velocity (maintaining E/A ratio and DT), ESV, wall stiffness and SI from their respective baseline values. Peak E-wave velocity contributes the most to Γ_I_, with a percentage increase of 151.6%, which is far higher than the other factors. Wall stiffness causes the most significant effect on V_p_ (−30%), followed by peak E-wave velocity (14.4%), ESV (−9%) and SI (3%). On the other hand, IVPD increases with an increase in the peak E-wave velocity (80.3%) and wall stiffness (17%), but decreases with increasing SI (−30.9) and ESV (−14.3%). EDP and vMS follow similar trends, with peak E-wave velocity contributing the most (431.5% and 371.6%), followed by wall stiffness (113.1% and 149.8%), ESV (−83% and −73.4%) and SI (13.3% and 28.9%). With regards to peak strain rates, an increase in the peak E-wave velocity causes a substantial increase in the strain rates (SR_L_: 140.7%, SR_C_: 131.1%, SR_R_: 80.0%). In contrast, increasing ESV significantly decreases strain rates (SR_L_: −44.5%, SR_C_: −46.4%, SR_R_: −28.0%). SI (SR_L_: 12.3%, SR_C_: 9.1%, SR_R_: 3.7%). SI and wall stiffness, on the other hand, have negligible effects on strain rates.

### III. Effect of Infarct Location

**Figure 10:**
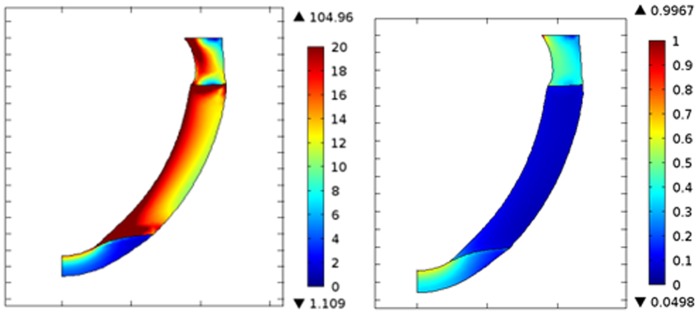
von Mises stress (left) and total strain (right) distribution for ischemic DCM (lateral infarct) during end of the filling phase.

With regards to the intraventricular flow distribution (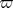, 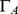, 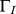 and 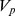), similar results were obtained for ischemic DCM with apical and lateral infarction. As illustrated in Figure 10, for ischemic DCM with lateral infarction, much lower strain levels were observed at the mid LV region as compared to other regions along the myocardial wall. Moreover, as for the apical infarction case, high wall stress was apparent at the border zone between the ischemic and the non-ischemic regions in the ischemic DCM condition with lateral infarction.

**Figure 11:**
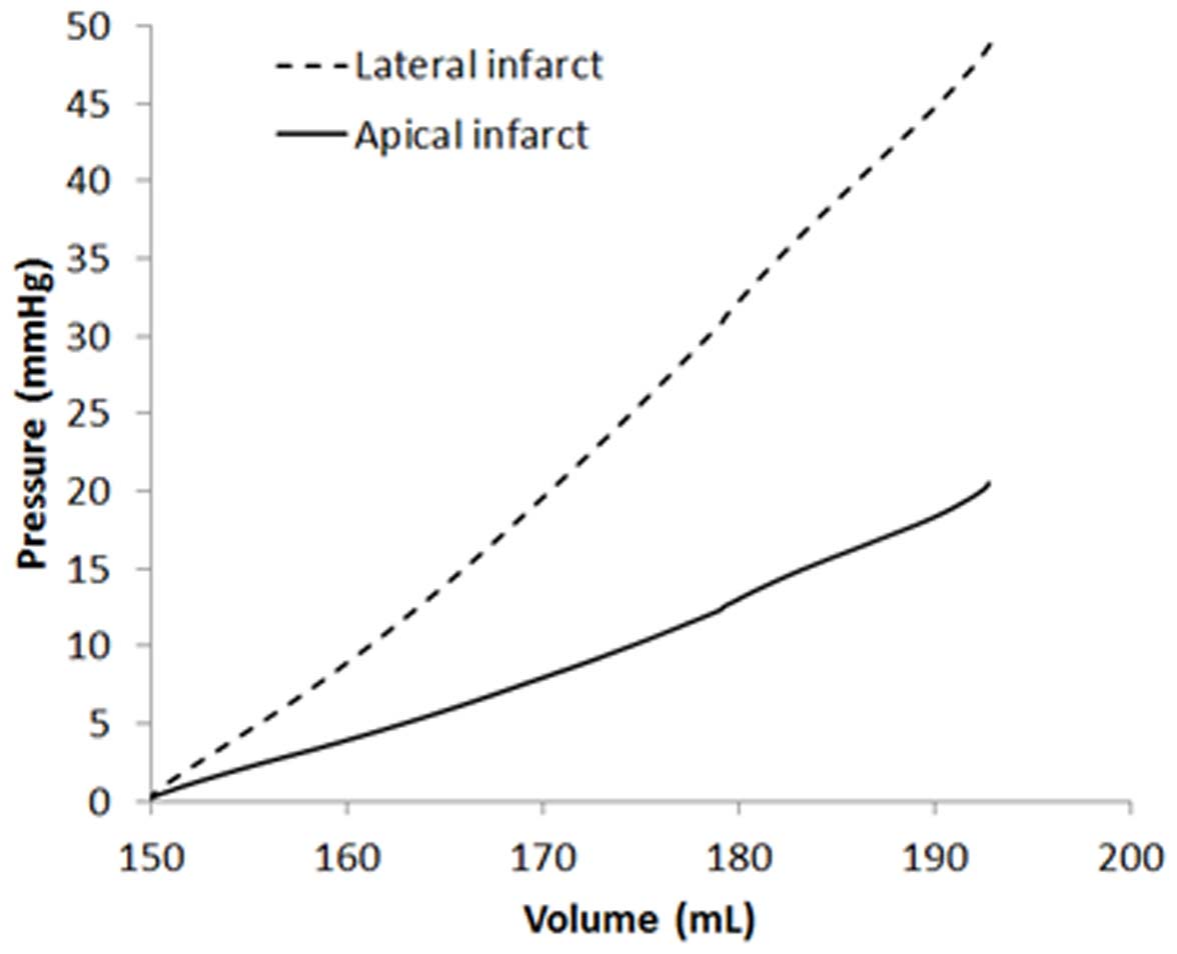
Pressure-volume relationship for the ischemic DCM cases (apical infarction and lateral infarction) throughout the filling phase.

Conversely, Figure 11 shows that a change in the infarct location significantly alters the PV relationship. Despite having the same material properties for both cases (apical vs. lateral infarct), the PV curve of the ischemic DCM condition with lateral infarction is much steeper, reflecting a stiffer myocardial wall. This results in a much higher end diastolic pressure at the same inflow velocity, which may indicate worse prognosis in these patients.

## Discussion

The abnormal flow pattern in diseased LV is often associated with thrombus formation where a flow stasis region promotes blood clot formation and contributes to low flow propagation velocity [30], [31]. Thrombus formation, particularly at the apical region of the LV, has frequently been reported in patients with DCM [32]. It is believed that this is associated with low vortex intensity [11] and flow propagation velocity [21]. During the filling phase, the formation of a vortex ring aids in preserving the kinetic energy of the incoming flow through fast rotating motion [33] and induces blood exchange to prevent flow stagnation [31]. As the vortex ring rotates around its center of mass while translating from the base to the apex, the total kinetic energy of the incoming blood flow is converted into rotational and translational energy [34]. The translational energy allows penetration of the vortex ring to the stagnant flow region at the apex, while blood mixing is enhanced by the rotational energy.

Despite the importance of the vortex on intraventricular flow dynamics, not many studies have quantitatively analyzed vortex-related parameters in the LV computational model. While Loerakker et al. [11] observed a decreased vortex area and vortex intensity in a computational model of LV with DCM, opposite results were obtained in the Baccani et al study [21] which used the same filling velocity for both normal and DCM conditions. The above contradictory result can be explained by the present study, which shows that filling velocity is the prime determinant of vortex intensity due to its close relationship with kinetic energy. Conversely, an increase in the LV size as in [21], causes an increase in the vortex area and a minor increase in the vortex intensity, due to a larger space for growth of the vortex ring. Although we observed lower vortex intensity in the normal LV in our simulation results when compared to DCM with a restrictive filling pattern, a stronger vorticity field and thus higher average vorticity is obtained in the normal LV, due to a larger vortex-LV relative area. Furthermore, as the vortex approaches the compliant LV wall, it induces a substantial perturbation in the pressure field near the myocardial wall which causes a significant deformation of the wall [35], thereby preventing flow stagnation.

While flow propagation velocity (V_p_) has been proven to be a relatively preload-independent index of diastolic function through various clinical studies [7], its value is still debated in patients with hypertrophic [36] and restrictive DCM [37]. The present study reveals that V_p_ is substantially (30%) decreased by a twofold increase in wall stiffness; thus it forms a useful index for passive chamber compliance. This agrees with De Boeck et al. [38], who suggested that an increase in wall stiffness leads to an increase in pressure wave propagation velocity (principle of acoustic conduction) which in turn slows down the propagation of the velocity wave. On the other hand, a decrease in LV size (ESV) causes an increase in V_p_, as it provides a narrow path which directs the propagation of the flow velocity downwards. Our results may explain the high V_p_ values obtained in patients with hypertrophic LV. With regards to the effect of preload on V_p_, our results showed a positive correlation between peak E-wave velocity and V_p_. Upon closer investigation, we found that V_p_ increases exponentially with peak E-wave velocity, i.e. an increase in peak E-wave velocity causes a minor increase in V_p_ at low values, but significantly increases V_p_ at high velocity. Consequently, preload-independency may be weakened in patients with high E-wave velocity.

Generation of sufficient IVPD improves the efficiency of early diastolic LV filling [8]. Extensive studies have reported that the prime determinant of early peak IVPD is elastic recoil and myocardial relaxation rate [39]. In the present study, we found that early peak IVPD coincides with peak acceleration of the E-wave in all cases, whilst its magnitude correlates positively with the rate of change of the filling velocity, which is in turn dependent on the myocardial relaxation rate in a real clinical setting. On the other hand, an increase in ESV decreases peak early IVPD. Our results are in agreement with Yotti et al. [8], who proposed that early peak IVPD is influenced mainly by inertial acceleration (rate of change of the filling velocity) and convective deceleration which depends on the ventriculo:annular ratio.

With the availability of myocardial deformation imaging technology, diastolic strain rate, which characterizes the speed of myocardial deformation during the diastolic phase, has become increasingly popular for the assessment of diastolic function (active relaxation) [40] as well as the identification of myocardial infarct size and location [41]. Our simulation results agree with the published literature [17], [42], which report reduced longitudinal and circumferential diastolic strain rates in patients with DCM. A decrease in the diastolic strain rate leads to impaired filling efficiency and potentially reduces the contraction force during ejection. Since myocardial deformation (LV expansion) is directly related to the filling velocity, the peak E-wave velocity contributes the most to strain rates in all directions as compared to other factors. This is in agreement with clinical findings, which report that strain rate measurements are significantly dependent on preload [43]. A major advantage of the diastolic strain rate over filling velocity in the assessment of diastolic function is its ability to describe regional myocardial function. Our simulation results showed that strain rate along the infarcted region (apex) of the myocardial wall is substantially lower than other regions of the wall. This is in agreement with clinical findings [44], which reported significant correlation between early strain rate and regional stiffness, and proposed that segmental diastolic strain rate can indicate the presence and extent of regional ischemia [45].

With regards to end diastolic pressure (EDP) and average von Mises stress (vMS), our simulation results showed that both measures are substantially raised in the DCM conditions, mostly due to an increase in wall stiffness. An increase in ESV and sphericity index, which is associated with adverse ventricular remodeling, causes opposite effects on EDP and vMS, with the former decreasing EDP and vMS while the latter inducing an increase in both EDP and vMS. High stress is found near the border zone between the ischemic and the non-ischemic regions, as observed by Jackson et al. [46], and it is believed that this may further worsen myocardial function around this region.

### Limitations

In our simulations, the implementation of a fixed constraint at the basal location of the FSI boundary caused exceptionally high stress around that point as wall movement was restricted at the constrained boundary. This may result in inaccurate stress analysis at the basal region, especially near the constrained site. However, we have not found any improved implementation for this type of simulation [22].

The use of an axisymmetric ventricular model in this study may affect the pathway of blood flow and vortex propagation. An asymmetrical LV geometry or patient-specific model should be incorporated in future studies. In addition, the mitral valve was modeled as an opening orifice and non-moving structure in this study, therefore the effect of mitral valve opening/closing dynamics on the intraventricular flow was not taken into account.

The present model did not incorporate myocardial fiber orientations in the LV wall, which may affect regional strain and stress distributions along the LV. Furthermore, the model did not take into account active relaxation and elastic recoil, which are important during early diastole.

In the present study, model parameters and validation data were based on published literature instead of our own data. In order to ensure the robustness of the model, model parameters were sourced from many studies [8], [25]–[27] using a variety of medical imaging and clinical measurement techniques, which were independent of the studies used to validate the outcomes of the model [7], [17], [38], [42], [46].

### Conclusion

We have presented a 2D axisymmetric FSI model of the LV under normal and DCM conditions, and investigated the effect of DCM on intraventricular fluid dynamics and myocardial wall mechanics. Intraventricular flow dynamics and myocardial wall deformation was significantly impaired in DCM, with low vortex intensity, flow propagation velocity, IVPD and strain rates, but high end diastolic pressure and wall stress. Our sensitivity analysis showed that flow propagation velocity substantially decreases with an increase in wall stiffness, and is relatively independent of preload at low peak E-wave velocity. Early IVPD is influenced mainly by the rate of change of early filling velocity and ESV which changes the ventriculo:annular ratio. Regional strain rate, on the other hand, was significantly correlated with regional stiffness, and therefore forms a useful indicator for myocardial regional ischemia. The present work forms an important framework for the investigation of mechanisms leading to observable changes in patients with DCM which is important for the clinical management of these patients.
